# SARS-CoV2 Vaccination Adverse Events Trend in Italy: A Retrospective Interpretation of the Last Year (December 2020–September 2021)

**DOI:** 10.3390/vaccines10020216

**Published:** 2022-01-30

**Authors:** Nicola Di Fazio, Giuseppe Delogu, Giuseppe Bertozzi, Vittorio Fineschi, Paola Frati

**Affiliations:** 1 Department of Anatomical, Histological, Forensic and Orthopedic Sciences, Sapienza University of Rome, Viale Regina Elena 336, 00161 Rome, Italy; nicola.difazio@uniroma1.it (N.D.F.); giuseppe.delogu@uniroma1.it (G.D.); paola.frati@uniroma1.it (P.F.); 2 Department of Clinical and Experimental Medicine, University of Foggia, 71100 Foggia, Italy; giuseppe.bertozzi@unifg.it; 3 IRCSS Neuromed Mediterranean Neurological Institute, Via Atinense 18, 86077 Pozzilli, Italy

**Keywords:** vaccine, COVID-19, AIFA, pharmacovigilance, adverse effect, Italy

## Abstract

At the end of 2020, a vaccination campaign against COVID-19 was launched. In 2021, legal obligations for health workers, as well as specific regulations for all workers, were introduced. The global SARS-CoV-2 pandemic was followed by epochal changes in life, school, and work habits in Italy. Therefore, the pharmacovigilance work currently being conducted in Italy by the AIFA concerning the recording and analysing of adverse reactions related to the use of vaccines has proved to be very important. The latest report, including a period of 10 months from December 2020 to September 2021, has allowed us to combine the results received so far, and to compare the safety of all vaccines currently available in Italy. The results of this analysis are highly encouraging and reveal the statistical reliability of the safety of the COVID-19 vaccines currently used in Italy. The dissemination of these findings could increase the public’s awareness of vaccines and their ability to make free and informed choices concerning vaccination. The potential increase in the Italian population’s adherence to the vaccination campaign could ultimately be a decisive factor in achieving herd immunity and the final resolution of the pandemic.

## 1. Introduction

In December 2019, a new strain of the SARS-CoV species had its first outbreak in the Chinese province of Hubei [1] and soon spread rapidly worldwide, causing the World Health Organization (WHO) to declare pandemic status in March 2020 [2]. Since Italy identified its first case in the Lombardy Region, the country has seen a very rapid diffusion of the virus. The 9th of March 2020 has become a day of historical interest: the Italian Government implemented extraordinary measures to limit viral transmission, and, for the next two months, the whole country was subject to a state of lock-down [3]. After several epidemiological and political developments, at the end of 2020 the European continent began a vaccination campaign against the virus. Italy officially started its campaign on 31st December, giving priority to frail population groups and healthcare workers [4,5]. Moreover, to obtain the highest compliance possible among the latter group, vaccination became compulsory for Italian health professionals in April 2021 [6,7]. Although this law has never been extended to other non-medical areas of work, in July 2021 a Labor Territorial Court issued an order which granted employers the right to suspend the service and pay of employees who refuse to undergo vaccination [8]. Such regulations are aimed at achieving the highest possible compliance of the eligible Italian population, which, 10 months after the vaccination campaign began, had a vaccination rate of 82% relating exclusively to the eligible administration group. This rate, when put in a European and global context, can be considered positive [9]. However, the imposition of vaccination against COVID-19 has generated mixed feelings among the Italian public, demonstrating its vulnerability to disinformation like never before [10]. Therefore, collecting pharmacovigilance data about the anti-COVID-19 vaccination campaign could be a very important task in transmitting real and scientifically proven information on the safety of these active substances; or, on the contrary, highlight any critical issues in this area [11].

Periodically, the Italian Drugs Agency (Agenzia Italiana del Farmaco, AIFA) has released surveillance reports focused on COVID-19 vaccines’ adverse reactions in Italian territory. The last edited report, which spanned the first ten months of the Italian vaccination campaign (27 December 2020–26 September 2021) [12], will be the subject of the current paper. The National Drug Adverse Event Reporting Network will be a key focus because, as a result of the preliminary findings of the three-stage testing [13], it allows the recording of a considerable amount of information about a given drug. [14] In the marketing phase of the vaccine, the administration of the active substance to more or less heterogeneous population groups allows the acquisition of a larger amount of data, and is burdened by a lower risk of bias than the experimental phase [15]. In this way, the safety of a drug can be significantly increased, and its potential suitability for certain patient groups can be investigated [16,17]. Both doctors and citizens can report on the vaccine [18]. Nevertheless, a key step remains in reconstructing the causal link between the drug and the event. This crucial task is entrusted to the Global Advisory Committee on Vaccine Safety (GACVS), which is a specially trained body belonging to the World Health Organization (WHO) [19]. When the causal link is not present, the case will be classified as an adverse event. If, on the other hand, there is a causal link, it will be necessary to identify two further sub-phases. If the event has had a harmful effect, it will be classified as an adverse reaction; if the event has not had a harmful effect, it will be called an undesirable effect.

## 2. Considerations on Vaccination Data for the Period December 2020–September 2021

From the analysis of the data collected for the period December 2020–September 2021, it is possible to deduce that the reporting rate, which is about 120 reports per 100,000 doses administered, has a higher concentration in the intermediate age groups (30–59 years); the lower rate recorded belongs to the category of the population under twenty years of age.

It is also possible to state that the percentage of “non-serious” events is greater than 85%; in the remaining category, most cases are on the way to recovery. Finally, it emerges that the pattern of reports, regardless of the type of schedule and type of vaccine, shows a more homogeneous trend than that estimated in previous months.

The data presented are based on more than 84 million administered doses. Most of these consist of the compound Comirnaty. However, this number requires interpretation, as the reports show different incidences depending on whether the vaccine administered is a first dose, a second dose (completion of the cycle), or a third dose (i.e., a “booster”, which is expected in certain cases). For instance, 2/3 of the total reactions occurred following the administration of the first dose of the vaccine, whereas out of the 46,000 third doses administered, only one non-serious report was received. This trend can be observed in detail in Table 1, which is also stratified for each type of vaccine.

**Figure 1:**
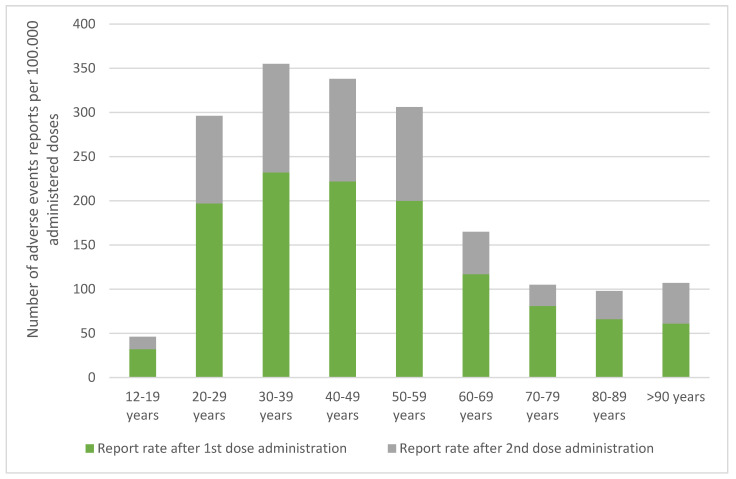
Adverse events report rate distribution per age range and administered dose (number of reports per 100,000 doses administered in the period 27 December 2020–26 September 2021 modified from Rapporto sulla Sorveglianza dei vaccini COVID–19 [12]).

As already mentioned, intermediate age groups are most affected by adverse events, while there is a lower reporting rate in two categories of individuals: those under the age of 20 and those over the age of 60 (Figure 1).

For the 12–19 age group, an adverse event rate of 24 per 100,000 doses was reported. A percentage of 23.1% of these was found to be “serious”. However, this overall number of adverse events is too limited to estimate the representativeness of the data. Most of these reactions, in any case, concern male subjects at the second dose, with symptomatology consisting of fever, headache, joint pain, and, less frequently, myocarditis/pericarditis.

In general, however, the distribution by type of event of all reports of subjects aged between 12 and 19 years is comparable to that of other age groups, regardless of the type of vaccine, the type of dose, and the intensity of symptoms.

**Figure 2:**
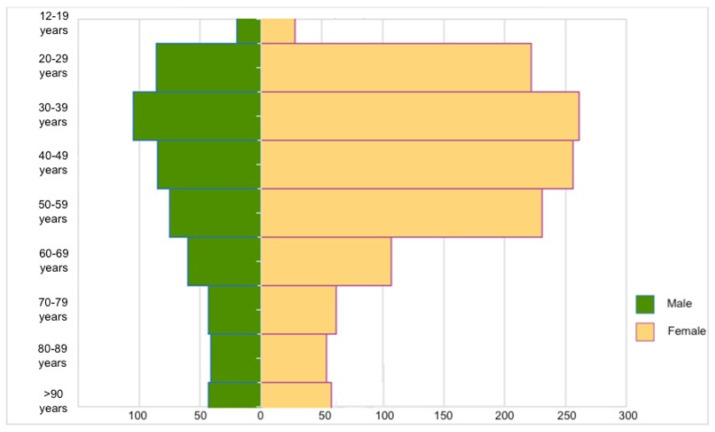
The distribution of the numbers of adverse events per 100,000 doses administered by sex and age group during the period 27 December 2020–26 September 2021 included in the National Pharmacovigilance Network (modified from Rapporto sulla Sorveglianza dei vaccini COVID-19 [12]).

It is also interesting to note that in all age groups, while there is substantial equivalence between the two sexes as regards the number of doses administered, the majority of reactions affected female subjects (71%) (Figure 2).

In addition, the reports were analyzed based on the employment of the person who made the report. About 68% of signalers appeared to be healthcare workers (of these, physicians comprised a percentage of 37.1%), while the percentage of other citizens signaling was 31.5%.

**Figure 3:**
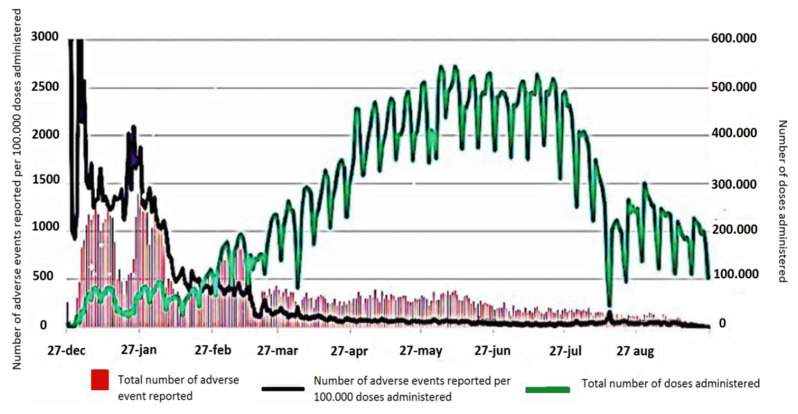
The trends of the numbers of doses administered and adverse events reported, and the estimate of the numbers of adverse events per 100,000 doses administered in the period 27 December 2020–26 September 2021 (modified from Rapporto sulla Sorveglianza dei vaccini COVID-19 [12]).

Although there has been a decline expected in the number of reports in the summer, the increasing rate of reports from the category of citizens appears to be encouraging (Figure 3).

It is also interesting to analyze the time latency with which the report was made. Analysis of the data shows that most adverse reactions occur in the short term: 48% of reports are made within the first 24 h and 76% are made within the first 48 h, while only 8% are carried out after one week of administration.

In addition, it should be noted that “serious” reactions also follow this temporal trend, occuring mostly after a short time from the administration of the dose.

**Figure 4:**
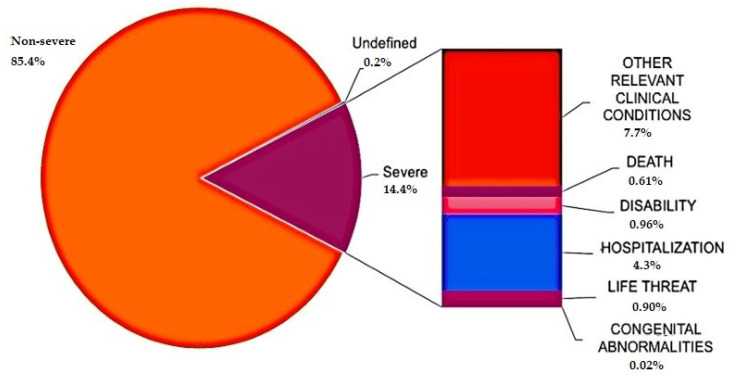
Distribution of the percentages of adverse events by severity included in the period 27 December 2020–26 September 2021 (the severity criterion is not indicated in 0.02% of the reports) (modified from Rapporto sulla Sorveglianza dei vaccini COVID-19 [12]).

In terms of the type of adverse reactions, it has already been stated that only 14.4% of the total can be defined as “serious” reactions; a further stratification of this category is available in Figure 4.

Of these serious reactions, 53.6% had a complete resolution or an improvement, whilst among the nonserious reactions, about 70% had a complete resolution or improvement. The deaths have resurfaced in the remaining cases. These events, equal to 608 so far, are intended as data available at the time of reporting or acquired subsequently in follow-up, with a maximum recorded interval of 189 days from vaccination. The incidence rate of overall deaths, below 1/100,000 doses, is characterized by a trend comparable to that observed in previous months. Additionally, in this case, there is a preponderance of the event “death” following the first dose of vaccine (397 cases following the first dose vs. 211 following the second dose). The average age of deaths, however, is equal to 76 years, with an almost complete correlation between the sexes.

There is a piece of strong evidence that no anaphylactic or allergic reaction has so far led to the death of a patient, while in most cases death is due to aggravation of pre-existing chronic diseases. A significant number of deaths have occurred as a result of the administration of the Comirnaty vaccine (391 events). However, Comirnaty being the most used vaccine, its death rate is necessarily reduced and is the lowest of all vaccines used (0.65/100,000 doses administered). The highest death rate, conversely, was recorded by the Janssen vaccine, with a value of 1.65 deaths per 100,000 doses. The overall figures are given in Table 2.

However, the assessment of the causal link between these reports and the vaccination event should be considered. A special algorithm developed by the WHO was applied in 71.5% of cases, with the result that only 16 cases out of 435 (or 3.7%, equal to 1 death per 5 million doses administered) were causally correlated with vaccination.

## 3. Individual Vaccine-Specific Results

Following a rapid general review of the data examined, a description of the main findings related to the individual active substance can be proposed. It should be reiterated an objective reading of these data must take into account the fact that the Comirnaty vaccine, introduced at the end of 2020 [20], constituted the only compound available for about 20 days, until the distribution of the Moderna (later renamed Spikevax) vaccine began [21]. Comirnaty is still the most widely used vaccine, so a comparison of adverse events requires the use of standardized rates. In addition, the Janssen vaccine being the most recent addition to the vaccine landscape (April 2021), and with only 1.5 million units of it having been administered, the scientific evidence available for this vaccine may be less statistically significant than the other vaccines [22].

Finally, it is appropriate to classify the compounds numbered according to their mechanism of action and category of patients to whom it was administered [23]: Comirnaty is a mRNA vaccine administered to individuals aged 12 years or older; Spikevax is a mRNA vaccine administered to individuals aged 12 years or older; Vaxzevria is a recombinant viral vector vaccine administered to individuals aged 18 years or older; Janssen is a viral vector vaccine administered to individuals aged 18 years or older.

### 3.1. Comirnaty

In a framework of substantial equivalence with the data of the previous months, most of the events reported for this compound concerned the category of general diseases (fever, asthenia, headache, paresthesia, arthro-myalgia, nausea, vomiting, and diarrhea) and reactions to the inoculation site. With four reports of “severe” reactions per 100,000 doses administered, the percentage of these events is 12%. Of these, moreover, only 3% have the specific wording “life-threatening”. It is worth noting that within the adverse reactions of special interest, there are six reported instances of myocarditis/pericarditis, three anaphylactic reactions, and two facial paralyses per million doses administered. Finally, a full resolution of the event was recorded in 52% of cases.

### 3.2. Spikevax

Similar to what was observed for the Comirnaty vaccine, the compound Spikevax presents a stable trend, with prevalent reactions of a general and reactive nature to the injection site. The percentage of serious reactions, in this case, is equal to 18.1% (about 3 per 100,000 doses administered), of which 5% of the type is “life-threatening”. Reactions of special interest may include, per million doses administered, 11 myocarditis/pericarditis, 2 anaphylactic reactions, and 2 facial paralyses per million doses administered. The fact that this compound belongs to the same category as Comirnaty reflects, at least in part, the homogeneity found in pharmacovigilance. In 38% of these cases, complete remission was required.

### 3.3. Vaxzevria

The principles set out for the previous two vaccines also apply to Vaxzevria. The percentage of “serious” reactions, amounting to 18%, is this time recorded in 11 cases every 100,000 doses administered, of which 5.8% are classified as “life-threatening”. From the point of view of the reactions of special interest, two anaphylaxis, one acute or subacute neuropathy (Guillain-Barrè syndrome), and one atypical thrombotic event (intracranial) associated with thrombocytopenia can be counted per million doses administered. The full resolution rate for this compound is 44%.

### 3.4. Janssen

As already mentioned, this is the last vaccine introduced. The scarcity of currently available data, although already present in previous reports, seems to show a framework already settled and similar to what was claimed for the previous active ingredients. The majority of the recorded adverse events, therefore, must be traced back to the sphere of the general pathologies and the reactions to the site of inoculum. With 5 reports of “serious” events for every 100,000 reports, moreover, this category was equal to 24% of all the reactions. It should be noted, however, that this figure derives from only 71 reports received so far, which is an insufficient number from which to construct an estimate. It is possible, however, to state events of special interest, such as atypical thrombotic episodes associated with platelets thrombocytopenia. At present, acute neurological or anaphylactic neurotics are less than 1 per million doses administered. A further reflection of this condition is the percentage of complete remissions from adverse reactions, which is only 22%; however, a further 41% are associated with “improvement of clinical conditions”.

### 3.5. Heterologous Vaccination

Since September 2021, due to contingency factors, a total of 640,000 individuals under the age of 60 have completed the vaccination cycle consisting of two doses using different compounds. Specifically, they were people who had been inoculated with a first dose of the Vaxzevria vaccine, followed by a dose of either Comirnaty (76% of cases) or Spikevax (24%). With a general reporting rate of 40 adverse events per 100,000 doses, the percentage of adverse events did not differ between the two groups of vaccinated subjects with different compounds, or concerning the data already stated from single schedules. It should be noted, however, that in this case the reports were made in 52% of cases by citizens, and only in 46% of cases by health workers.

## 4. Discussion

Based on the data contained in the Italian AIFA reports, it is possible to state that the current trend of reports of adverse events related to vaccination against COVID-19 in Italy is highly encouraging in terms of the safety of currently available compounds. The limited timescale in which pharmaceutical companies have been required to produce such vaccines has raised many doubts among the public, which have been further exacerbated by reports of particular adverse events, including cerebral venous sinus thrombosis [24,25]. In this context, the news from the latest AIFA report is particularly encouraging. The trend of adverse reactions to vaccination against COVID-19 is completely acceptable from the clinical and epidemiological point of view, as expected from preliminary studies [26]. Furthermore, the large number of patients subjected to vaccination has makes the data collected statistically significant, an essential requirement for the interpretation of any scientific element. It has already been highlighted in this paper that a potentially important aspect of this reporting system is linked to the fact that most of the reports came from health professionals, suggesting that an important portion of (mostly minor) episodes was lost because they may have gone unreported by the citizens. The reversal of this trend, observed in the field of heterologous vaccination (52% of reports from ordinary citizens) and probably dictated by the apprehension generated by the media pressure concerning “serious” reactions, has produced results that are completely comparable to those collected in previous months [27]. It is therefore conceivable that the percentages currently published are reliable and representative of the risk associated with the administration of these vaccines [28]. For reasons related to the number of doses delivered, the question of the actual safety of the compound Janssen is still pending. However, Janssen shows preliminary results in line with other vaccines, and therefore, in the absence of any divergent developments, it can be considered equally clinically safe.

It is also interesting to compare the Italian data with the European data made available by the ECDC (European Centre for Disease Prevention and Control) [29] on the rate of adverse reactions from vaccination against COVID-19. It is essential to proceed with two considerations immediately. First, the vaccines taken into account in the ECDC report are the same as those analyzed by the AIFA and reported in this paper. Second, the most recent ECDC report considers adverse reaction rates to be limited to the period from 27 December 2020 to 28 April 2021, and therefore to the first administrated dose. The European data, like the Italian data, confirm that the administration of the first dose leads to a higher rate of adverse events than the administration of subsequent doses. Therefore, it can be claimed that the Italian rate of reporting, limited to the period of the first 4 months of the vaccination campaign, is comparable to the European framework. This value is already sufficient to consider the reliability of the data provided by the AIFA.

In addition, given the linear trend that the reports have shown in the 10 months of observation in Italy, we can cautiously pronounce a safety judgment about the active ingredients currently used for the anti-COVID-19 vaccination campaign. However, further months of observation appear necessary in order to make a final judgment on the safety of anti-COVID-19 vaccination.

## 5. Conclusions

In conclusion, at such an exceptional and delicate historical moment, the prompt availability of vaccines for the fight against the SARS-CoV-2 pandemic has necessitated an accurate study of their actual safety. In the light of the results of the latest AIFA report, which concerned the first 10 months of pharmacovigilance, it can be said that the positive results of pre-commercialization clinical studies have been confirmed by the application of these active substances to the population. Therefore, the risk associated with the administration of these vaccines can be considered to be fully commensurate and acceptable. In the meantime, more transparency in communication about compensation procedures for vaccine side effects is necessary for those countries in which the compensation for COVID-19 vaccine-related adverse events is not in actuality foreseen [6]. On 1 December 2021 the AIFA Technical and Scientific Committee, accepting the opinion expressed by the European Medicines Agency (EMA), approved the extension of the indication for use of the Comirnaty vaccine, in the specific formulation of 10 mcg/dose, for the age group 5–11 years. Therefore, in Italy, it is now possible to proceed with the inclusion of this age group in the SARS-CoV-2/COVID-19 vaccination programme, taking into account the priority order of groups, particularly the “highly fragile” category [30].

Finally, the emergence of these figures could make it possible to correctly target the unvaccinated population, facilitating them in making conscious and informed choices, and thereby contributing decisively to obtaining that heard immunity so agonized for in order to secure a return to the forms of public and private life that characterized the pre-COVID era [31,32,33].

## Figures and Tables

**Table 1:** Distribution of adverse event reporting rates by dose number (number of reports per 100,000 doses administered in the period 27 December 2020–26 September 2021, modified from Rapporto sulla Sorveglianza dei vaccini COVID-19 [12]).

Vaccine	1st Dose Adverse Event Reporting Rate (Number of Reports per 100,000 Doses Administered)	Confidence Interval at 95%	2nd Dose Adverse Event Reporting Rate (Number of Reports per 100,000 Doses Administered)	Confidence Interval at 95%	Cumulative Adverse Event Reporting Rate (Number of Reports per 100,000 Doses Administered)	Confidence Interval at 95%
Comirnaty	137	136–138	89	92–94	114	113–115
Spikevax	104	101–107	62	58–63	84	82–86
Vaxzevria	320	316–324	28	22–24	185	183–187
Janssen	92	87–97	-	-	92	87–97
All vaccines	158	157–159	77	76–78	120	119–121

**Table 2:** Distribution of the number of deaths after vaccination by product in the period 27 December 2020–26 September 2021 (modified from Rapporto sulla Sorveglianza dei vaccini COVID-19 [12]).

Vaccine	Fatal Cases	Deaths Rate per 100,000 Administered Doses
Comirnaty	391	0.65
Spikevax	96	0.91
Vaxzevria	98	0.81
Janssen	23	1.56
TOTAL	608	0.72

## Data Availability

The data that support the findings of this study are openly available in “Rapporto Sulla Sorveglianza dei Vaccini COVID-19 (9) 27/12/2020–26/09/2021.” at https://www.aifa.gov.it/documents/20142/1315190/Rapporto_sorveglianza_vaccini_COVID-19_9.pdf (accessed on 4 December 2021).
